# Seroprevalence of Sandfly‐Borne Phleboviruses Belonging to Three Serocomplexes (*Sandfly fever Naples*, *Sandfly fever Sicilian* and *Salehabad*) in Dogs from Greece and Cyprus Using Neutralization Test

**DOI:** 10.1371/journal.pntd.0005063

**Published:** 2016-10-26

**Authors:** Sulaf Alwassouf, Vasiliki Christodoulou, Laurence Bichaud, Pantelis Ntais, Apostolos Mazeris, Maria Antoniou, Remi N. Charrel

**Affiliations:** 1 UMR “Emergence des Pathologies Virales” (EPV: Aix-Marseille Univ - IRD 190 - Inserm 1207 - EHESP), Marseille, France; 2 Institut hospitalo-universitaire Méditerranée infection, APHM Public Hospitals of Marseille, Marseille, France; 3 Veterinary Services of Cyprus, Nicosia, Cyprus; 4 Laboratory of Clinical Bacteriology, Parasitology, Zoonoses and Geographical Medicine, Faculty of Medicine, University of Crete, Voutes. Heraklion. Crete, Greece; The Faculty of Medicine, The Hebrew University of Jerusalem, ISRAEL

## Abstract

Phleboviruses transmitted by sandflies are endemic in the Mediterranean area. The last decade has witnessed the description of an accumulating number of novel viruses. Although, the risk of exposure of vertebrates is globally assessed, detailed geographic knowledge is poor even in Greece and Cyprus where sandfly fever has been recognized for a long time and repeatedly. A total of 1,250 dogs from mainland Greece and Greek archipelago on one hand and 422 dogs from Cyprus on the other hand have been sampled and tested for neutralising antibodies against Toscana virus (TOSV), Sandfly fever Sicilian virus (SFSV), Arbia virus, and Adana virus i.e. four viruses belonging to the 3 sandfly-borne serocomplexes known to circulate actively in the Mediterranean area. Our results showed that (i) SFSV is highly prevalent with 71.9% (50.7–84.9% depending on the region) in Greece and 60.2% (40.0–72.6%) in Cyprus; (ii) TOSV ranked second with 4.4% (0–15.4%) in Greece and 8.4% (0–11.4%) in Cyprus; (iii) Salehabad viruses (Arbia and Adana) displayed also substantial prevalence rates in both countries with values ranging from 0–22.6% depending on the region and on the virus strain used in the test. These results demonstrate that circulation of viruses transmitted by sand flies can be estimated qualitatively using dog sera. As reported in other regions of the Mediterranean, these results indicate that it is time to shift these viruses from the "*neglected"* status to the "*priority"* status in order to stimulate studies aiming at defining and quantifying their medical and veterinary importance and possible public health impact. Specifically, viruses belonging to the Sandfly fever Sicilian complex should be given careful consideration. This calls for implementation of direct and indirect diagnosis in National reference centers and in hospital microbiology laboratories and systematic testing of unelucidated febrile illness and central and peripheral nervous system febrile manifestations.

## Author Summary

Phleboviruses transmitted by sandflies are endemic in the Mediterranean basin. An increased number of new viruses was described during the last decade. However, levels of exposure of human and animal populations are poorly known. A total of 1,250 dogs from Greece and 422 dogs from Cyprus were tested for the presence of neutralising antibodies signing previous infection with selected phleboviruses representing the 3 serological complexes known to be present in the Old World: Toscana virus (TOSV), Sandfly fever Sicilian virus (SFSV) and Salehabad viruses (Arbia and Adana viruses). Our data showed that (i) SFSV is largely predominant with infection rates higher than 50%, (ii) TOSV is widely distributed with 4.4% and 8.4% in Greece and Cyprus, respectively, and (iii) that viruses belonging to the Salehabad serocomplex should be further studied for their capacity to cause human disease in view of prevalence rates in dogs up to 22.6%. These findings confirm that dogs can be considered as excellent sentinels for sandfly-borne phleboviruses. The results also underline the importance to study the role of SFSV in humans and may lead to the set-up diagnostic tests for patients presenting unexplained febrile illness and neuroinvasive infections. Further studies are also needed to define whether these viruses cause diseases in dogs.

## Introduction

In the Old world, phleboviruses (*Bunyaviridae* family, *Phlebovirus* genus) transmitted by phlebotomines consist of three species or antigenic groups, namely Sandfly fever Naples, Salehabad, and Sandfly fever Sicilian serocomplexes. Each species contains several viruses among which Naples, Sicilian and Toscana virus cause 3-day fever, commonly called sandfly fever in humans; Toscana virus (TOSV) causes of neuroinvasive human infections such as meningitis and encephalitis [1].

In Greece, outbreaks of sandfly fever were reported in Athens among the local population, and among American, British and German troops during World War II [2]. Sandfly fever has been described in Cyprus and Greece with both sporadic cases and epidemics [3–7]. In both countries, the high rates of antibodies observed in seroprevalence studies indicate that viruses belonging to Sandfly fever Naples and Sandfly fever Sicilian serocomplexes are transmitted by local sand flies to human populations [7–10].

Sandfly fever Cyprus virus (SFCV), closely related to Sandfly fever Sicilian virus (SFSV), was isolated during a large outbreak of sandfly fever in Swedish United Nations troops stationed in Cyprus; few cases were also caused by TOSV [5]. In Greece, in recent sporadic cases of meningitis, (i) TOSV RNA was detected in the CSF of a patient [11], and (ii) viral RNA corresponding to Adria virus, a novel virus belonging to the *Salehabad* species, was also identified in the CSF [12, 13]. To date, SFSV or another SFS-like virus have been neither isolated nor detected by molecular techniques in Greece.

During the last decade, field-to-laboratory integrated studies associating virologists, parasitologists and entomologists have discovered several new phlebotomine-borne phleboviruses; thus there is an increased diversity in each of the three aforementioned species or serocomplexes [14]. Although the pathogenicity of most of these newly discovered viruses remains unknown, they are sympatric with recognized pathogenic phleboviruses [15–17]. Because several viruses of the same serocomplex co-circulate in various regions, interpretation of seroprevalence studies requires using techniques that hold the capacity to discriminate between these antigenically-related viruses. To the best of our knowledge, all studies performed in Greece and Cyprus used either ELISA or IFA tests, which are notoriously prone to cross-reactivity between viruses belonging to the same serocomplex [7–10]. To conduct our nation-wide (mainland Greece, Greek islands, Cyprus) seroprevalence study in dogs, we selected neutralisation tests which is the most discriminant assay as previously reported in Algeria, Tunisia, Turkey and Portugal [15, 18–20]. Although virus exposure to viruses may be quantitatively different in humans and dogs, because of different feeding preferences of phlebotomines, recent studies suggest that virus circulation can be estimated using either human or dog sera since dogs live in close proximity to humans and are readily infected by these viruses [15, 18, 20, 21]. In our study, dog sera were tested for the presence of neutralising antibodies against TOSV, SFSV, and two viruses belonging to the Salehabad complex (Arbia virus isolated in Italy and Adana virus isolated in Turkey).

## Materials and Methods

### Animal and samples

From 2005 to 2010, a total of 422 and 1,250 dog sera were collected in Cyprus and Greece, respectively. These sera originated from the five districts of Cyprus and 32 prefectures belonging to 12 regions of Greece (Table 1).

**Table 1:** Characteristics of dogs tested in the study.

Region	N° of sera	Prefecture	N° of sera	Male	Female	Ratio M/F	Median age
Attica	410	Attica	410	79	331	0.24	24
Central Macedonia	129	Chalkidiki	62	36	26	1.38	60
		Imathia	4	3	1	3.00	42
		Kilkis	4	4	0	-	99
		Serres	26	11	15	0.73	60
		Thessaloniki	32	16	16	1.00	54
		Veria	1	1	0	-	16
Crete	199	Chania	89	40	49	0.82	42
		Heraklion	51	23	28	0.82	48
		Lassithi	33	16	17	0.94	36
		Rethymno	26	19	7	2.71	27
East Macedonia and Thrace	91	Drama	16	8	8	1.00	36
		Evros	35	17	18	0.94	60
		Kavala	21	15	6	2.50	30
		Rodopi	19	9	10	0.90	24
Epirus	37	Arta	30	24	6	4.00	27
		Ioannina	7	5	2	2.50	18
Ioanian Islands	79	Corfu island	79	49	30	1.63	48
North Aegean	81	Chios island	15	7	8	0.88	54
		Lesvos Island	66	33	33	1.00	48
Peloponesse	60	Argos	3	1	2	0.50	96
		Arkadia	56	26	30	0.87	48
		Korinthia	1	1	0	-	36
South Aegean	30	Cyclades	19	12	7	1.71	60
		Dodecanese	11	6	5	1.20	24
Sterea Hellas	119	Evia	110	69	41	1.68	48
		Fokida	1	0	1	-	30
		Fthiotida	5	2	3	0.67	30
		Viotia	3	2	1	2.00	48
Thessaly	1	Trikala	1	0	1	-	48
West Greece	14	Aitoloakarnania	1	5	8	0.63	48
		Achaia	13	1	0	-	60
**Total Greece**	**1250**	** **	**1,250**	**540**	**710**	**0.76**	**36**
Ammochostos	67			36	31	1.16	24
Larnaca	27			12	15	0.80	36
Limassol	97			47	50	0.94	36
Nicosia	74			31	43	0.72	33
Paphos	177			76	101	0.75	24
**Total Cyprus**	**442**	** **	** **	**202**	**240**	**0.84**	**36**

Veterinarians were asked to provide dog samples from animals visiting their clinic for any reason: vaccination, hair cut, nail cut, deworming, general check up, treatments, and other purposes, without discrimination. The animals were examined clinically and peripheral blood samples (without EDTA) was collected, after the written consent of the owner, and questionnaires with personal, epidemiological, and clinical data for each dog were completed.

Only domestic dogs that were raised in the area were considered for the study. The domestic dogs were included after owners’ informed consent. Information regarding age, sex, was obtained after interviewing dog owners (Table 1). Each dog was examined clinically by the veterinarian and blood samples were collected. Whole blood samples were collected (1–2 mL) by cephalic or jugular venipuncture and serum was separated by centrifugation and stored at −20°C.

Data on the region, gender, and age (distributed according to 3 classes: young 6–11 months, adult 12–83 months, senior ≥ 84 months) were recorded. This study was ethically approved by the Institutional Animal Care and Use Committee of the University of Crete Medical School and conform with the European Union Directive 2010/63/EU regarding use of animals and biological specimens in research, as well as the relevant Hellenic legislation (Presidential Decree 160/91, under the Code Numbers 31 EE 05, 31 EPR 04 and 31EP 020). Written informed consent was obtained from the dog owners, according to the aforementioned national legislations.

### Virus microneutralisation assay (MN)

Sera were tested by the virus microneutralisation assay (MN), described for phleboviruses [19] in parallel for 3 distinct sandfly-borne phleboviruses: (i) TOSV strain MRS2010-4319501 (TOSV belongs to the *Sandfly fever Naples virus* species or complex) [22], (ii) SFSV strain Sabin [23], (iii) Arbia-like virus strain T131 (*Salehabad* species or complex), and (iv) Adana virus strain 195 (*Salehabad* species or complex) [15].

Briefly, two-fold serial dilutions from 1:10 to 1:80 were prepared for each serum and a volume of 50μL was pipeted into 96-well plate. Viruses were titrated in Vero cells (ATCC CCL81). A volume of 50 μL containing 1000 TCID_50_ was added into each well except for the controls that consisted of PBS. A volume of 50 μL of EMEM medium enriched with 5% fetal bovine serum, 1% Penicilin Streptomycin, 1% L-Glutamine 200 mM, 1% Kanamycin, 3% Fungizone, was added to each well of the controls.

The plates were incubated at 37C° for one hour. Then, a 100μL suspension of Vero cells containing approximately 2 x10^5^ cells/mL of EMEM medium (as previously described) was added to each well, and incubated at 37C° in presence of 5% CO2.The first row of each plate contained control sera diluted 1:10 and Vero cells without virus.

After 5 days (Toscana and Arbia virus) and 7 day (Sicilian and Adana virus), the microplates were read under an inverted microscope, and the presence (neutralization titer at 20, 40, 80 and 160) or absence (no neutralization) of cytopathic effect was noted. Cut-off value for positivity was set at titre ≥ 20 [15, 18, 20, 21].

Due to insufficient volume in Greek samples, ADAV was used for testing Cyprus specimens, only.

### Mapping

Dog seroprevalence for each virus was estimated for each prefecture and mapped using the geographical information system software (GIS, Redlands, CA; ArcGIS 10).

### Statistical analysis

The chi-square or Fisher’s exact tests were used to compare percentages of positivity among categories of the same independent variables and also the total prevalence of each virus. A p value < 0.05 was considered as statistically significant. Analyses were performed with StatLib and SPSS® 21 software for Windows.

## Results

### Sera collection and characteristics of the animals

In Greece, a total of 1,250 sera (540 male and 710 female, sex ratio 0.76) were collected. The median age was 36 months (range: 3–216). The sera were collected from 32 prefectures, but owing to the variability in the number of collected sera from each prefecture (range: 1–410), the sera were grouped into 12 regions. Of these 12 regions, Thessaly was not included in the analyses because it consisted of 1 serum only. For the other 11 regions, the number of sera ranged from 14 to 410.

In Cyprus, a total of 442 sera (202 male and 240 female, sex ratio 0.84) were collected. The median age was 36 (range: 3–144).They consisted of 67, 27, 97, 74, and 177 sera collected from the districts of Ammochostos, Larnaca, Limassol, Nicosia, and Paphos, respectively.

The two dog populations had the same median age (36 months) and a similar sex ratio (0.76 *vs* 0.84).

Characteristics of the dogs and their geographic origin are presented in Table 1.

### Microneutralising antibodies (NT-Abs)

Results for domesticated dogs living in Greece and in Cyprus are presented in Table 2 and Table 3, respectively.

**Table 2:** Seroprevalence of 1,250 dog sera from Greece.

Greece	N° of tested sera	N° of interpretable sera	TOSV		SFSV		ARBV	
			*N° of Pos (%)*	*P value*	*N° of Pos (%)*	*P value*	*N° of Pos (%)*	*P value*
**Region**				**0.248**		***<0*.*001***		***0*.*013***
Attica	410	404	20 (5)		343 (84.9)		2 (0.5)	
Central Macedonia	129	123	9 (7.3)		97 (78.9)		8 (6.5)	
Crete	199	183	2 (1.1)		100 (54.6)		5 (2.73)	
East Macedonia and Thrace	91	81	2 (2.5)		56 (69.1)		4 (5)	
Epirus	37	37	2 (5.4)		27 (73)		1 (2.7)	
Ionian islands	79	77	3 (3.9)		47 (61)		0 (0)	
North Aegean	81	75	4 (5.3)		38 (50.7)		5 (6.7)	
Peloponnese	60	52	3 (5.6)		40 (76.9)		2 (3.9)	
South Aegean	30	25	0 (0)		16 (64)		1 (4)	
Sterea Hellas	119	114	5 (4.4)		77 (67.5)		3 (2.6)	
Thessaly	1	1	0 (0)		1 (100)		0 (0)	
West Greece	14	13	2 (15.4)		10 (76.9)		0 (0)	
**Total**	**1,250**	**1,185**	**52 (4.4)**		**852 (71.9)**		**31 (2.6)**	
**Gender**				**0.887**		***0*.*003***		***0*.*029***
Female	710	676	29 (4.3)		509 (75.3)		12 (1.8)	
Male	540	509	23 (4.5)		343 (67.4)		19 (3.7)	
**Total**	**1,250**	**1,185**	**52 (4.3)**		**852 (71.9)**		**31 (2.6)**	
**Age group (months)**				**0.373**		**0.236**		**0.632**
[6–11]	36	33	1 (3)		21 (63.6)		0 (0)	
[12–83]	1,010	960	39 (4.1)		700 (72.9)		26 (2.7)	
[≥ 84]	204	192	12 (6.3)		131 (68.2)		5 (2.6)	
**Total**	**1,250**	**1,185**	**52 (4.4)**	** **	**852 (71.9)**	** **	**31 (2.6)**	** **

**Table 3:** Seroprevalence of 442 dog sera from Cyprus.

Cyprus	N° of tested sera	N° of interpretable sera	TOSV		SFSV		ARBV		ADAV	
			*N° of Pos (%)*	*P value*	*N° of Pos (%)*	*P value*	*N° of Pos (%)*	*P value*	*N° of Pos (%)*	*P value*
**District**				0.094		***<0*.*001***		0.280		***0*.*011***
Ammochostos	67	57	0 (0)		15 (26.3)		1 (1.8)		2 (3.5)	
Larnaca	27	20	1 (5)		8 (40)		1 (5)		4 (20)	
Limassol	97	70	8 (11.4)		43 (61.4)		4 (5.7)		8 (11.4)	
Nicosia	74	58	4 (6.9)		37 (63.8)		1 (1.7)		9 (15.5)	
Paphos	177	164	18 (11)		119 (72.6)		13 (7.9)		37 (22.6)	
**Total**	**442**	**369**	**31 (8.4)**		**222 (60.2)**		**20 (5.4)**		**60 (16.3)**	
**Gender**				0.259		0.241		0.648		0.323
Female	240	198	20 (10.1)		125 (63.1)		12 (6.1)		36 (18.2)	
Male	202	171	11 (6.4)		97 (56.7)		8 (4.7)		24 (14)	
**Total**	**442**	**369**	**31 (8.4)**		**222 (60.2)**		**20 (5.4)**		**60 (16.3)**	
**Age group (months)**				0.499		0.912		***0*.*018***		***0*.*023***
[6–11]	25	22	1 (4.6)		13 (59.1)		1 (4.6)		2 (9.1)	
[12–83]	382	317	26 (8.2)		192 (60.6)		14 (4.4)		48 (15.1)	
[≥ 84]	35	30	4 (13.3)		17 (56.7)		5 (16.7)		10 (33.3)	
**Total**	**442**	**369**	**31 (8.4)**	** **	**222 (60.2)**	** **	**20 (5.4)**	** **	**60 (16.3)**	** **

Toxic activity in the serum was detected in 65 and 73 sera from Greece and Cyprus, respectively; therefore calculations were done on the basis of 1,185 and 369 sera of Greece and Cyprus, respectively. As previously shown [26, 27], a cut-off titre ≥ 20-when used for 1000TCID_50_ inoculum, is equivalent to a cut-off titre ≥ 40 when a 100 TCID_50_ is used [28].

**Fig 1:**
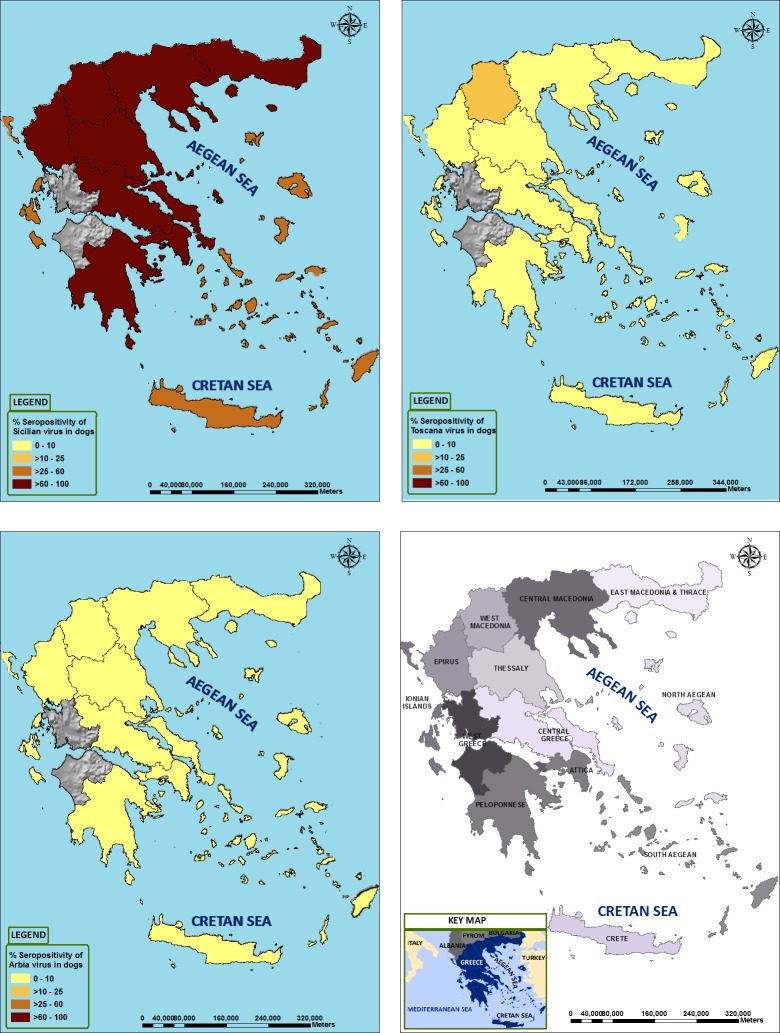
Geographic distribution of neutralising antibodies against Toscana virus (panel A), Sandfly fever Sicilian virus (panel B), Arbia virus (panel C) in Greece, using ArcGIS 10). Panel D represent the localisation of regions listed in Table 2.

**Fig 2:**
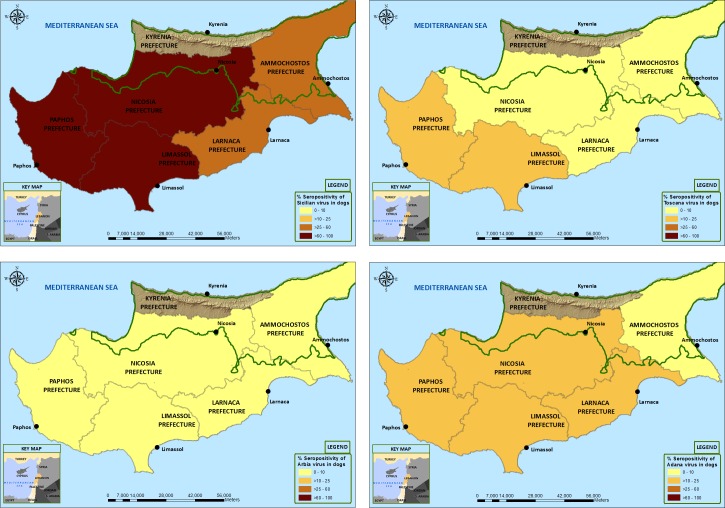
Geographic distribution of neutralising antibodies against Toscana virus (panel A), Sandfly fever Sicilian virus (panel B), Arbia virus (panel C), and Adana virus (panel D) in Cyprus, using ArcGIS 10.

In Greece, a much higher rate of SFSV-NT-Ab was observed (71.9%) compared with 4.4% and 2.6% for TOSV and ARBV, respectively (Table 1 and Fig 1). Similar results were observed in Cyprus where SFSV-NT-Ab was present in 60.2% of the dog sera, whereas 16.3%, 8.4% and 5.4% of sera were positive for ADAV, TOSV and ARBV, respectively (Table 2 and Fig 2).

The distribution of TOSV positive sera is quite homogenous within the studied regions (p = 0.248, p = 0.094). There is no significant difference according to the sex of the dogs. In contrast, it appears that the prevalence increases with the age, although it is not statistically significant even when the results of dogs from Greece and Cyprus are merged (3.7% / 5.3% / 7.7%, p = 0.3).

In both countries, a statistic association was found between SFSV prevalence and geographic area.

In Cyprus, none of the sera containing ARBV-NT-Abs were also positive for ADAV-NT-Abs and vice versa; this demonstrated that there is no cross-reactivity through MN assay between these two viruses despite the fact that they belong to the same serocomplex. Exposure to ARBV and ADAV is significantly associated with the age with higher rates observed in older dogs. In contrast, the high rates of SFSV-NT-Abs were observed in the 6-11-month age class in Cyprus and Greece.

## Discussion

At the outset of our study, the following data were available for Greece: (i) there was no serological data in domestic animals (cattle, goats, sheep, dogs or cats) for any phlebovirus transmitted by sand flies, (ii) in the 1970's, 13.1% of 38 adults living in Crete had NT-Abs against Naples virus, and 24.7% and 8.5% of 632 human sera from Athens inhabitants had NT-Abs against Naples virus and Sicilian virus, respectively [29]; (iii) more recent studies reported various rates of TOSV IgG using ELISA and/or IIF tests in continental Greece as well as in the Ionian and Aegean islands [8–10]; (iv) TOSV RNA (belonging to the lineage C) was detected in the CSF of a patient with meningitis [11], and (v) viral RNA corresponding to Adria virus, a novel virus belonging to the *Salehabad* species, was also identified in the CSF of a patient with meningitis [12, 13]. To date, SFSV or another SFS-like virus have been neither isolated nor detected by molecular techniques in Greece.

At the outset of our study, the following data were available for Cyprus: (i) there was no serological data in domestic animals (cattle, goats, sheep, dogs or cats) for any phlebovirus transmitted by sand flies; (ii) first evidence of the presence of TOSV, Naples and Sicilian viruses were observed in Swedish soldiers of the United Nations force [3] through detection of NT-Abs and isolation of strains of Naples and Sicilian viruses [30]; (iii) NT-based seroprevalence results showed that Naples, Sicilian, and TOSV were endemic with respective rates of 57%, 32% and 20% [7]; (iv) investigation of a second outbreak in Greek troops stationed in Nicosia of which almost 50% developed febrile syndrome had resulted in isolation of Cyprus virus (SFCV), closely related but distinct from Sicilian virus although belonging to the SFSV serocomplex [4, 5].

The recent discovery of several new viruses belonging to the three species associated with phlebotomines in the Old World has raised questions about the viral strain currently circulating in the two regions. Since broadly cross-reactive techniques such as ELISA and IIF are not capable to distinguish between viruses belonging to the same serocomplex, we decided to use microneutralisation assay using viral strains or surrogates which presence had been assessed in Greece and Cyprus. Indeed, we consider it valid to use SFSV as a surrogate for SFCV (isolated in Cyprus) and other SFSV-related viruses because amino acid distances observed between the proteins that elicit neutralizing antibodies (Gn and Gc) are well within the acceptable range, ie <5% different for SFSV and SFSV-related viruses[25, 31]. Thus, neutralising antibodies are unlikely to discriminate between closely-related SFSV isolates.

Since collecting human sera displaying a large geographic distribution was challenging, we decided to use dog sera; indeed, dog sera can be good surrogates for the following reasons: (i) dogs are readily infected with phlebotomine-borne phleboviruses which are human pathogens [18, 20, 31, 32]; (ii) domestic dogs live in close contact with humans and therefore are exposed to sandfly bites, although different feeding preferences of sand fly species have to be considered [31].

The largest amount of data available on dogs, at the outset of this study, concerned TOSV, which observed rates (4.4% in Greece and 8.4% in Cyprus) are in the same order of magnitude as those recently reported in dogs in Tunisia (6.8%, [20]), in Algeria (4.3%, [18]), in France (3.9%, [21]) and in Portugal (6.8% [31]. Because all these studies measured neutralising antibodies against TOSV, their results are comparable and they reflect local circulation of TOSV only, not other viruses belonging to the SFNV complex. Together these results demonstrate that TOSV can readily infect dogs. Exposure level of dogs and humans may be drastically different in the same area as previously shown in Tunisia where MN-based seroprevalence rates were respectively at 6.8% in dogs compared with 41% in humans [20].

In the present study, dogs living in the Ionian island of Corfu showed a much lower seroprevalence compared to the human population living in the same island (3.9% *vs* 51.7%) [8]; however, in this case the techniques used were different; in the human study, ELISA/IIF detected not only TOSV IgG but also IgG raised after infection with other viruses belonging to the *Sandfly fever Naples* serocomplex in which 6 new viruses were described during the last decade (Arrabida, Fermo, Granada, Massilia, Punique, Zerdali) [16, 33–37] in addition to Naples virus (a proven human pathogen) and Tehran virus. Although none of these viruses were detected or isolated in Greece or Cyprus, the presence of one of these 6 recently discovered viruses or of a yet to be discovered virus may account for these apparently discrepant results. Last, these techniques do not hold the same sensitivity [38]. The same explanation applies for discrepancies observed between high rates of ELISA/IFA TOSV IgG reported in Aegean islands (17.6%,11.5%, 20%, 22% and 34.7% for Lesbos, Rodos, Siros, Crete and Evia, respectively) [9] compared with our findings: 5.3% in north Aegean islands (Chios and Lesbos), 0% in south Aegean islands (Rodos, Siros and Santorini), 1% in Crete and 4.4% in Evia (Stere Hellas)(Table 2). In Central Macedonia, 7.3% of dog sera contained TOSV-NT-Abs, which is in agreement with reported cases of human infections [38, 39] and a recent study showing that TOSV and/or antigenically related viruses are circulating extensively in the area [10]. It is worth underlining that, despite using the same technique, discrepant prevalence rates were also described between dogs and humans in Tunisia [19, 20]. Therefore, it is difficult to compare results of serological studies performed with different techniques. When using the same technique, results observed in humans and in dogs consistently detected TOSV although they varied considerably quantitatively; therefore dogs can serve as sentinel for humans and vice versa for assessing the presence of TOSV although quantitative results must be interpreted carefully.

The absence of cross-protection between ARBV-NT-Abs and ADAV-NT-Abs confirm previous data from Turkey [15]. Accordingly, cumulative percentage of viruses belonging to the *Salehabad* species is 21.7%. This is congruent with the results observed in Adana, southern Anatolia, Turkey where domestic animals were presenting high rates of NT-Ab against viruses belonging to the *Salehabad* serocomplex [15]. Tesh et al [29] did not detect NT-Abs against Salehabad virus (SALV) in human populations suggesting that SALV was not infecting humans. In contrast, NT-Abs against Medjerda Valley virus were described in 1.35% (14/1260) of human sera collected from the general population living in Northern Tunisia [24]. This suggests that at least some viruses belonging to the *Salehabad* complex can infect humans and other vertebrates. Interestingly, Adria virus RNA has been detected in the CSF of a Greek patient presenting with meningitis [13] but was never isolated precluding serological studies aiming at defining the possible impact of this virus in the region and beyond. However, molecular detection of Adria virus in Albania (in sand flies) and in Greece (in human) suggests that its distribution might cover a large geographic area. This constituted the first direct evidence supporting human pathogenicity of a virus belonging to the *Salehabad virus* complex. Isolation of Adria virus is now a priority in order to pursue the studies using neutralization-based serological studies in humans and animals.

Very high rates of SFSV-NT-Abs were observed in Cyprus and Greece. In the latter, rates were consistently above 50% (range 50.7–84.9%); in Cyprus, rates were above 40% (range 40.0–72.6%) except in Ammochostos (26.3%). The extremely high prevalence rates observed with SFSV in young dogs show that this virus continues to circulate very actively in these regions, and beyond as recently described in dogs from Tunisia (50.8%, [20]) and in Portugal (38.1%, [31]). In both countries, a statistic association was found between SFSV prevalence and geographic area. The differences of prevalence depending upon the region may be due to the geographic and climatic characteristics of these regions which affect the distribution, proliferation and abundance of phlebotomine vectors of SFSV. Analysis of the questionnaires did not identify any clinical manifestations such a fever and/or neurological signs during the past weeks and months in the SFSV-positive dogs. This tends to suggest that SFSV is not or mildly affecting dogs during the viremic period. Whether or not dogs can play a role as reservoir in the natural cycle remains to be studied. To do so, experimental studies to understand the virus kinetics are necessary. Also, studies aiming at the identification of viremic domestic dogs should be planned in high prevalence areas.

The massive prevalence of SFSV-NT-Ab observed in our study is not unexpected and is congruent with entomological and human data in the literature: (i) isolation of Corfu virus on the eponymous island from *Phlebotomus neglectus* [17]; (ii) SFSV IgG detected by IFA in human sera in Northern Greece (Macedonia), Central Greece (Evritania and Larisa), North–Western Greece (Epirus), and Corfu Island; (iii) detection of Chios virus, SFSV-like, in Chios island; (iv) sandfly fever epidemics were reported in Swedish UN soldiers and Greek soldiers in 1984 and 2002, respectively [3–5]; (v) a high attack rate (63%) in tourists hosted in Cyprus for a short period [6]; (vi) a 32% prevalence rate of SFSV IgG in Cyprus native population [7]. In contrast with the two other serocomplexes which display an important range of genetic distance between their respective members, Sicilian virus strains are genetically and antigenically much more closely related [14, 16]; therefore, exposure to different SFSV strains (Italy, Turkey, Cyprus, Greece, Ethiopia) can be measured by using the prototypic Italian strain. Despite high rates of antibodies in humans and other vertebrates and successive outbreaks in Italy, Cyprus, Greece and Ethiopia [3, 5, 23, 40], SFSV remains a neglected pathogen, almost never included in diagnostic algorithms despite repeated and accumulating evidence of its involvement in febrile syndromes and in neuroinvasive infections.

In conclusion, this study indicates that (i) sandfly-borne phleboviruses belonging to 3 distinct genetic and antigenic groups are widely spread and co-circulate; (ii) dogs represent excellent qualitative sentinels for virus transmitted by sandflies and further studies must be done to estimate the role of dogs in the dynamics of transmission, and whether they play a role as reservoir hosts in the natural cycle of these viruses.

Since several of these viruses are proven human pathogens, our results plead for performing similar studies using human sera to identify geographic hot spots. The increasing number of sequence data for these phlebotomine-borne phleboviruses now enables to design and develop real-time molecular assays. The improved diagnostic toolbox will allow to investgate the medical impact of these viruses in patients presenting unexplained febrile illness and neuroinvasive infections.
